# Single Tracks as a Key Factor in Additive Manufacturing Technology—Analysis of Research Trends and Metal Deposition Behavior

**DOI:** 10.3390/ma13051115

**Published:** 2020-03-03

**Authors:** Justyna Patalas-Maliszewska, Eugene Feldshtein, Oleg Devojno, Małgorzata Śliwa, Marharyta Kardapolava, Nikolaj Lutsko

**Affiliations:** 1 Institute of Mechanical Engineering, University of Zielona Góra, Prof. Z. Szafrana 4, 65-516 Zielona Góra, Poland; E.Feldsztein@ibem.uz.zgora.pl (E.F.); M.Sliwa@iizp.uz.zgora.pl (M.Ś.); 2 Faculty of Mechanical Engineering, Belarusian National Technical University, Khmelnitsky str., 9, build. 6, 220013 Minsk, Belarus; devoino-o@mail.ru (O.D.); margokardo@mail.ru (M.K.); nilucko@tut.by (N.L.)

**Keywords:** additive manufacturing trends, single-track features, Bayes algorithm, Ni-based alloy, Fe–Al bronze, single and double composite layers

## Abstract

In recent years, general studies on Selective Laser Melting (SLM)/Selective Laser Sintering (SLS)/direct metal deposition (DMD) technologies, as well as studies on detailed issues in this area, have been carried out. However, a research gap is observed in investigations into the features of single tracks in the above-mentioned technologies. On the basis of data published in 2016–2019, an approach was adopted for a preliminary quantitative analysis of the knowledge base and also trends observed in the development of new technologies. This study demonstrates the effectiveness of the data mining technique based on the Bayes algorithm for analyzing trends in processes of additive manufacturing and the practical application of the knowledge received using the Bayes algorithm. After the analyses referred to above were completed, single and double layers of a composite material based on the Ni-based alloy and Fe–Al bronze were analyzed under different processing conditions. The effects of laser spot speeds and pitches on microhardness, microstructure, and interlayers’ features were described. So, the innovative approach, namely, the combination of the analysis of the scientific database of the phenomenon under study and the subsequent experimental investigation of its features, is the scientific novelty of the present study.

## 1. Introduction

Additive manufacturing (AM) is one of the newest technologies used in the production of machine parts. Its main principle is the formation of finished parts basing on computer three-dimensional models. Powders or filaments are used in such technologies. A precision three-dimensional computer model with the use of high-precision computerized laser systems practically eliminates even the finish machining, and additionally allows us to make the parts much lighter and with more complex shapes than traditional technologies allow. All this relates primarily to the production of metal or ceramic parts, which are much more expensive and complex than parts made from polymers and polymer-based composites. AM is used in the aerospace industry, oil and gas industries, in shipbuilding, automotive, in the manufacture of medical products, etc. Its effectiveness can be estimated from the change in the known ratio between the masses of the workpiece and the part (buy to fly ratio). In traditional technologies for aircraft engines, it is equal 10:1 and in general engineering it is equal to 20:1, whereas for AM it is equal about 1:1, as Bhavar et al. described [1]. The ideal controllability of the process allows the formation of lattice structures with low density, but at the same time with high strength, good thermal properties, and a high capacity of the energy absorption.

Two main indicators characterizing any technology of the AM of metal parts are a type of source material (powder or filament) and an energy source (laser or electron beam). The AM technological processes can be divided into two groups: technologies based on the powder bed fusion (PBF) and technologies based on the direct metal deposition (DMD). In the first case, the thermal energy required for the fusion of particles is concentrated in selected regions of the continuous layer of powder (bed). In such processes, the powder material is applied to the previous, fused layers using various devices. In the other case, the source powder materials are point-fed directly into the zone of action of the laser beam, where they are melted.

In the case of laser technologies, characteristic representatives of the PBF methods are Selective Laser Melting (SLM) and Selective Laser Sintering (SLS), and also, independently, the DMD method. The technology selection depends on the chemical composition and properties of the powder used.

In recent years, general studies on SLM/SLS/DMD technologies, as well as studies on detailed issues in this area, have been carried out and a different apparature was used for this. In this research, data published from 2016 to 2018 to investigate the features of single tracks in SLM/SLS/DMD processing were used. Regarding tests using SLM machines, Guo et al. [2] tested a Nb-37Ti-13Cr-2Al-1Si (at%) alloy, and a series of single tracks and single layers were manufactured. Shrestha et al. [3] scanned single tracks of a Ti-6Al-4V alloy. Aversa et al. [4] researched the properties of Al-based LPBF single-scan tracks, and Rashid et al. [5] studied the characteristics of 316L stainless steel. Chao et al. [6] researched single-track depositions of Al0.3CoCrFeNi on a 253MA steel plate, while Criales et al. [7] presented a statistical modeling approach for forecasting the selected elements in the processing track of a Ni 625 alloy. Darvish et al. [8] focused their research on a CoCrMo alloy. 

Regarding studies involving the application of Nd:YAG lasers, Stašić, D. Božić [9] researched the effects of the main laser-cladding process elements on the structure of single tracks of 316L and 316L–NiB alloys. Ansari et al. [10] presented the results of the coaxial laser cladding of NiCrAlY powder on Inconel 738 superalloy. Cai et al. [11], described the manufacturing of the FeCoCrNiCux alloy using laser cladding. Du et al. [12] focused their research on a Ni-based material, and Nabhani et al. [13] focused their investigations on Ti-6Al-4V alloy. Barekat et al. [14] analyzed the effects of the main process parameters on the single tracks when laser cladding of Co–Cr–Mo alloy on γ-TiAl substrate. 

Using CO_2_ lasers, Seo and Shim [15] investigated issues in the formation of porous metallic materials using the laser-melting deposition of Ti6Al4V/TiH2 mixture. They discovered that each process parameter affects the mechanical properties of the material being produced. Wang et al. [16] focused their research on forming of an anomalous eutectic in a Ni–Sn alloy using laser cladding, and Devojno et al [17] studied the microstructure, microhardness, and tribological properties of an Al–Fe powder bronze single tracks and coating. 

Using high-power diode lasers, Fan et al. [18] studied the ceramics-based AM process, in particular, the effects of the energy density on the geometry, microstructure, and micromechanical properties of Al_2_O_3_ single tracks. Barroi et al. [19] evaluated the effects of the laser power on the shapes of single tracks based on LPBF single-scan tracks, and Riquelme et al. [20] researched the effects of the laser-cladding process parameters on the geometry and dilution of a ZE41 magnesium alloy. Arias-González et al. [21] studied the changes in the hardness of the microstructure and the composition of bronze coating deposited on AISI 4340 steel. Liu et al. [22] focused their research on modeling the geometry of a single-track cladding, and Lu et al. [23] studied the compositional and microstructural properties of a titanium oxide on Ti6Al4V substrate when laser deposition. Lei et al. [24] investigated the features of melt pool morphology for laser cladding. 

Using self-development machines, Nie et al. [25] analyzed the effect of the scanning speed on the formation of a single track during the SLM process for an Al–Cu–Mg alloy. Shi et al. [26] developed a method for improving the process parameters according to the melt-pool characteristics for production high-density TiAl parts. Wei et al. [27] studied the mechanical properties of an as-built Ti-5Al-2.5Sn-Ti alloy, and Zheng et al. [28] studied the effects of the process parameters and melt morphologies on the track features. Similarly, the Metelkova et al. [29] studied the features of the melt pool of 316L stainless steel, and Yang et al. [30] investigated the microstructural properties of a Ti6-Al4-V alloy. 

Bennett et al. [31] studied the impact of the main process parameters on the clad geometry and quality of an Inconel 718 superalloy, and Bax et al. [32] successfully carried out a systematic evaluation of the process parameter for laser cladding and directed energy deposition. Hybrid lasers were used in both investigations.

Using fiber lasers, Pariona et al. [33] performed simulation studies on the temperature and fluid-velocity field in a molten pool. In their research, laser-workpiece surface treatment of an Al-1.5 wt% Fe alloy with a single track was performed. Aboulkhair et al. [34] studied the single tracks from an Al alloy, and Zhou et al. [35] analyzed a Ni-based, single-crystal superalloy. Liu et al. [36] Chen et al. [37] focused on the geometric modeling of single-track and multi-track claddings deposited by a high-power diode laser with a rectangular beam spot or by experimental devices, respectively. Pei et al. [38] tested single tracks, densification, microstructure, and mechanical behavior of samples produced by selective laser melting of AlSi10Mg alloy, and Bertoli et al. [39] studied the morphology of a melt pool in which single tracks were created from 316L stainless steel powder. Yu et al. [40] investigated the changes in the mechanical properties of an YCF101 alloy, and Bailey et al. [41] examined the hardness properties of AISI H13 tool steel powder on H13 substrates. Arias-González et al. [42] studied the interactions between the processing parameters and the geometry of a single laser track of a Ni-based alloy deposited on cast iron. Kotoban et al. [43] compared SLM and DMD methods in producing single tracks of a Ni_3_Al intermetallic alloy. 

Tang et al. [44] focused on the simulation of the single-track formation of stainless steel using SLM. Xu et al. [45] researched the microstructure and mechanical properties of single tracks made of 316L stainless steel using a wave fiber laser and an arc welding machine. Ansari [10] studied the relationships between the process and geometric parameters of single clad tracks made from an Inconel 738 superalloy. Khairallah [46] studied the features of the melt pool using computer simulations, and Panwisawas et al. [47] modeled the effects of the process parameters on the microstructural properties of single tracks made of Ti-6Al-4V alloy. Liu et al. [48] developed a mathematical approach to study the effect of the substrate preset temperature on the microstructural properties, and Li et al. [49] examined the effects of the process parameters on the geometry and microhardness of single tracks deposited of an alumina ceramic.

In the context of Industry 4.0 concept, a manufacturing company can be competitive in the market owing to high-quality products and services it offers and the implementation of AM technologies [50]. Currently, research has been conducted on the possibilities of applying AM in Industry 4.0, and also in the application of materials in smart manufacturing [51], e.g., of alloys produced by AM processes [52]. This study was motivated by the need to establish the research trends in new technologies, particularly in single-track features in SLM/SLS/DMD processing, and to implement the results in experimental works. On the basis of the literature review and analyses of data published in 2016–2019, the features of single tracks in SLM/SLS/DMD processing were studied. A knowledge base was defined for further using the data mining technique, namely the Bayes algorithm. 

So, it can be concluded that one of the main research trends in the field of additive manufacturing of parts made of metals and alloys is the study of single layers features. This is a “common denominator” for elements based on steels, titanium alloys, chromium–nickel alloys, superalloys, ceramics, and a number of other materials. However, information on the use of smart materials, such as multi-components or composites, is almost completely absent. Besides that, it is very difficult to navigate through so widely available database and select actual research trends. 

There are different methods to analyze development trends in this area. The best-known are data mining techniques, i.e., analyses of statistical relationships and patterns in large scientific databases. Although the algorithms used for these purposes are very calculation intensive, the dynamic development of hardware technologies allows progress towards on-line data mining techniques. This approach extends the functionality of classic databases and enables the implementation of a new generation of applications for data analysis and decision support.

So, the first aim of this study is to demonstrate the effectiveness of the data mining techniques, namely the Bayes algorithm, for analyzing trends in features of new technologies, particularly single-track features in SLM/SLS/DMD processing. However, no less important is the practical application of the knowledge received using the Bayes algorithm. Thus, the other aim of this study is to analyze the possibility of depositing materials, namely the 12N-01 Ni alloy and the Fe–Al bronze, which were selected after the achievement of the first aim. Single and double layers of the composite material based on materials referred to above have been tested under different processing conditions.

Such a choice was made due to the fact that DMD technology is currently most promising. It has some advantages in comparison with traditional machining and coating technologies. Using DMD, work-piece properties can be improved by refining or combining materials. As a result of using this technology, the softer metal will have a hard, high-quality surface. Particularly, it is possible to combine a thermally insulating material with a material that resists high temperatures, salt, water, or chemicals. DMD technology secures rapid cooling rates, namely creates very low and controllable heat input with minimal dilution and heat effect zones as well as minimal stresses and distortions. Additionally, DMD results in faster production times and lower manufacturing costs due to minimizing or eliminating traditional machining technologies.

## 2. Principles of the Bayes Algorithm

Bayes algorithm was selected according to established rules, intended to formalize accumulated expert knowledge, in the following form: if the premises “are such”, the conclusion “is such.” The premise may contain a number of statements connected to logical functions [53]. The knowledge base for representing such knowledge includes a set of rules and facts. For such formalized expert knowledge, the Bayes algorithm is used to determine the level of accumulated knowledge [54]. In the Bayes algorithm, the occurrence of an event is directly related to its selected state (an alternative). The basis of the network work is the Bayes’ statement (1), which allows for updating the probability a priori, according to the observation of events [55]. This subsequently determines the probability of the a posteriori conditional event X, given information B:

(1) $$P(X|B)=\frac{P(B|X)P(X)}{P(B)}$$

P(X\B)—the a posteriori probability of event X, having obtained information B,
P(X)—the a priori probability of event X, before the appearance of information B,
P(B\X)—the probability of the appearance of information B, if X is true,
P(B)—the probability of the appearance of information B.

The Bayes network takes the form of an acyclic graph [56]; the nodes are variables, and the arcs (edges) are conditional relations. Unconnected nodes indicate conditionally independent variables. Dependent variables, which are related to probability functions, have an input set of values assigned to the preceding variables. Only direct dependencies are known and cited in the network. The “output” is the probability of the variable represented by this successor [57]. 

## 3. Trends in Single-Track Features in SLM/SLS/DMD Using the Bayes Algorithm

### 3.1. The Preliminary Quantitative Analysis of the Knowledge

**Figure 1:**
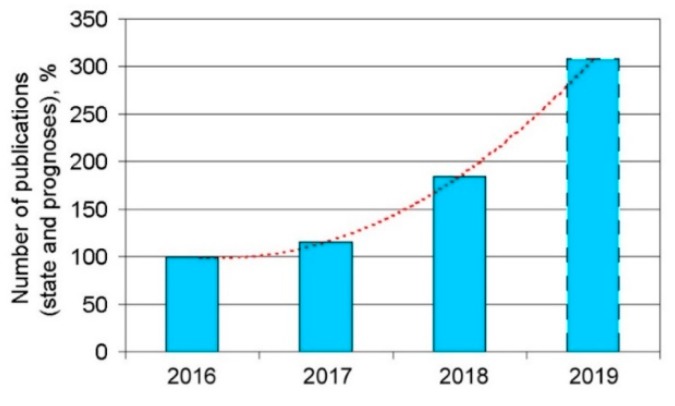
Increasing number of research articles containing expert knowledge on new technologies and involving investigations into single-track features in Selective Laser Melting (SLM)/LPBF processing.

**Figure 2:**
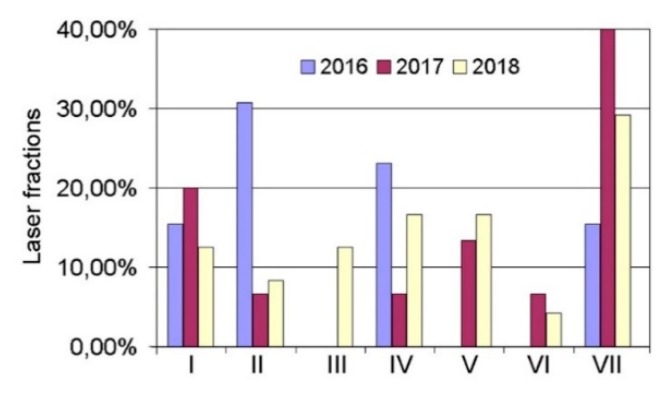
Frequency of use of laser types in research articles containing expert knowledge on new technologies and involving investigations into features of single tracks in laser processing (here, I— SLM machines, II—Nd:YAG lasers, III—CO_2_ lasers, IV—High-power diode lasers, V—Self-development machines, VI—Hybrid lasers, VII—Fiber lasers).

**Figure 3:**
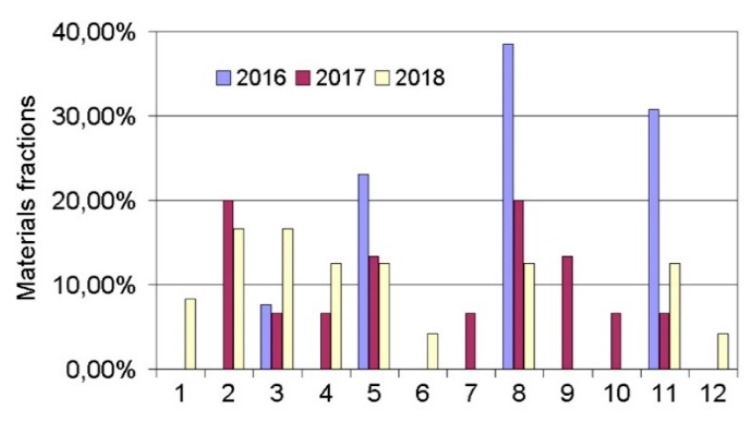
Frequency of use of materials in research articles containing expert knowledge of new technologies and involving investigations into features of single tracks in laser processing (here, 1—Fe alloys, 2—Ti alloys, 3—CrNi alloys, 4—Ceramics, 5—Al alloys, 6—High-speed steel, 7—Bronze, 8—Stainless steel, 9—Special alloys, 10—Tool steel, 11—Super-alloys, 12—others).

**Figure 4:**
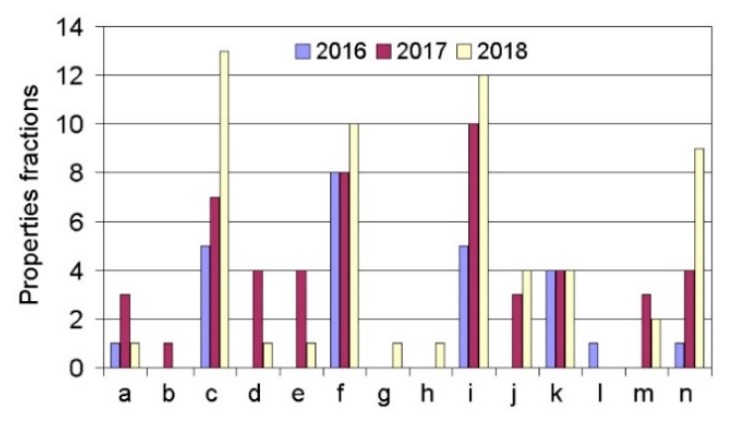
Frequency of the properties tested in research articles containing expert knowledge of new technologies and involving investigations into features of single tracks in laser processing (here, a—mechanical properties, b—structural properties, c—microstructure, d—microhardness, e—hardness, f—geometry, g—interlayer, h—wettability, i—morphology, j—porosity, k—features of melt pool, l—nano-hardness, m—cracking, n—modeling).

In the first stage of this research, the preliminary quantitative analysis of the knowledge stored above was performed. Increased interest in the subject was noted in 2018 (Figure 1). Moreover, it was found that in the research conducted, the fiber laser (Figure 2) was the most widely used type of instrument. According to the analysis of the expert knowledge contained in the research articles, it was suggested that research be performed on the following materials: Fe, Ti, Cr–Ni, ceramics, Al, high-speed steel, bronzes, stainless steels, special alloys, tool steel, superalloys, and others (Figure 3). The research was most frequently conducted on the following materials: Ti, Cr–Ni, Al, stainless steels, and superalloys. Furthermore, it was verified that the following properties were tested: the mechanical properties, structural properties, microstructure, microhardness, hardness, geometry, interlayer, wettability, morphology, porosity, features of the melt pool, nano-hardness, cracking, and modeling. In the research analyzed, the material used most often was powder (Figure 4).

### 3.2. Results of Bayes Algorithms Application

Thereafter, according to the main principles of the Bayes algorithm, the rule-based knowledge-representation method for knowledge base was used. 

If the research reported in a given article includes:

- the type of materials (powder or filament);
- the type of laser (SLM machines, Nd:YAG lasers, CO_2_ lasers, high-power diode lasers, self-development machines, hybrid lasers, or fiber lasers);
- the material (Fe, Ti, Cr–Ni, ceramic, Al, high-speed steel, bronze, stainless steel, special alloy, tool steel, superalloy, or YCF101 alloy);
- the properties tested (mechanical properties, structural properties, microstructure, microhardness, hardness, geometry, interlayer, wettability, morphology, porosity, features of melt pool, nano-hardness, cracking, or modeling).

Then, the value in the expert knowledge base is 1.

The rules for the impact factor (IF) of the journal in which the research article was located were also specified: if IF < = 2, then A; if 2 < IF < = 3, then B; if 3 < IF < = 4, then C; if 4 < IF < = 5, then D; if 5 < IF < = 6, then E; if IF < 6, then F.

**Figure 5:**
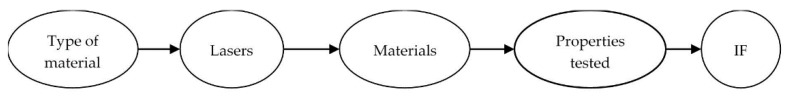
Bayesian network for expert knowledge.

It was said above that the Bayes algorithms were used for the formalized knowledge base. The relationships for a single track during laser cladding were modeled, which are denoted as BN < G, Θ >, where the set G contains net nodes, i.e., the process characteristics of the laser cladding: material type, laser type, materials, properties tested, and IF. The set Θ includes arches (edges) denoting the probability and conditional relationship between these nodes. The set G{Xi}, iϵN{1, …, 19} was defined. The set of edges was Θ{θn}, where θn = θX_i_ |πi = PBN (Xi |πi), demonstrating the probability of Xi conditioned on πi [58]. For each element of set G{Xi}, an alternative set x{xi,j} was assigned, j ϵ N. For nodes X1: type of material x ϵ {powder, filament}, X2: type of laser x ϵ {I, II, III, IV, V, VI, VII}, X3: material x ϵ {1, 2, 3, 4, 5, 6, 7, 8, 9, 10, 11, 12}, X4 to X18: “properties tested,” where x ϵ {0 “no” or 1 “yes”}, and X19: IF x ϵ {A, B, C, D, E, F}, according to the rules defined for the representation of expert knowledge. The dependencies among the characteristics of the processes of the studied set G (Figure 5) were proposed according to the expert knowledge accumulated.

Subsequently, a complete set of conditional probability distribution tables, which were obtained via learning, was implemented. A training set S = {S_1_, S_2_, ..., S_m_}, with m = {1, ..., 49}, included records composed of the parameters represented by set G, i.e., Xi and the alternatives assigned to these, x_i,j_. The ith sample contained S_i_ = {x_i_, 1, ..., x_i,j_}. The network was taught using the GeNie program. As a result, conditional probability tables (CPT) were defined for each Bayesian network node (Table 1). For example, if a “filament” type material was observed, the probability of using this in a “fiber laser” was P = 0,3482.

**Figure 6:**
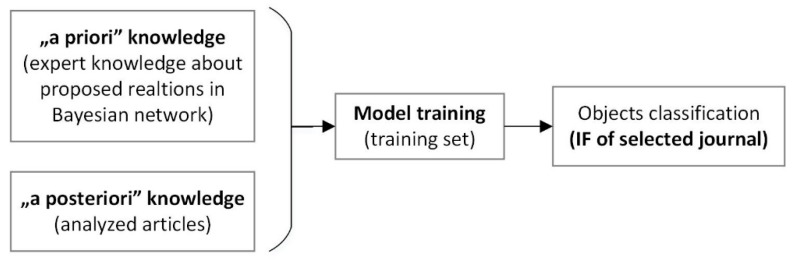
Learning Bayesian network for expert knowledge.

When determining the probability of an event P(X19: IF), the preceding vertices X1 to X18 were considered. According to (1), the Bayes algorithm was used to determine the level of expertise in laser technologies, particularly regarding the single track in laser cladding, while declaring the existence of identifying alternatives in the selected nodes (Figure 6). Theorem-type environments, including propositions, lemmas, and corollaries, can be formatted as follows.

**Figure 7:**
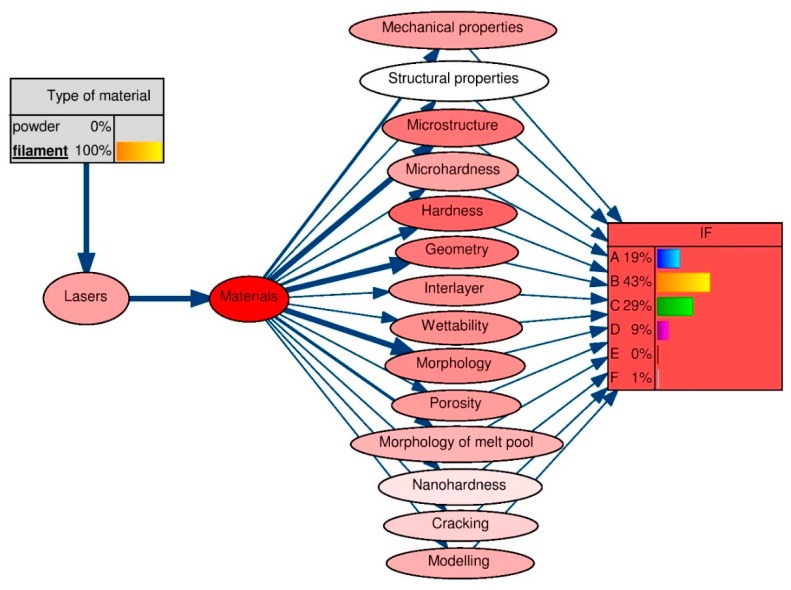
Expert knowledge levels of new technologies obtained via investigations into single-track features in laser processing, for studies performed using filament materials using the IF—the impact factor of the journal in which the research article was published. The level of expert knowledge in the journal with a specific IF index was: A = 19% (IF ≤ 2), B = 43% (2 < IF ≤ 3), C = 29% (3 < IF ≤ 4), D = 9% (4 < IF ≤ 5), E = 0% (5 < IF ≤ 6), F = 1% (IF > 6).

**Figure 8:**
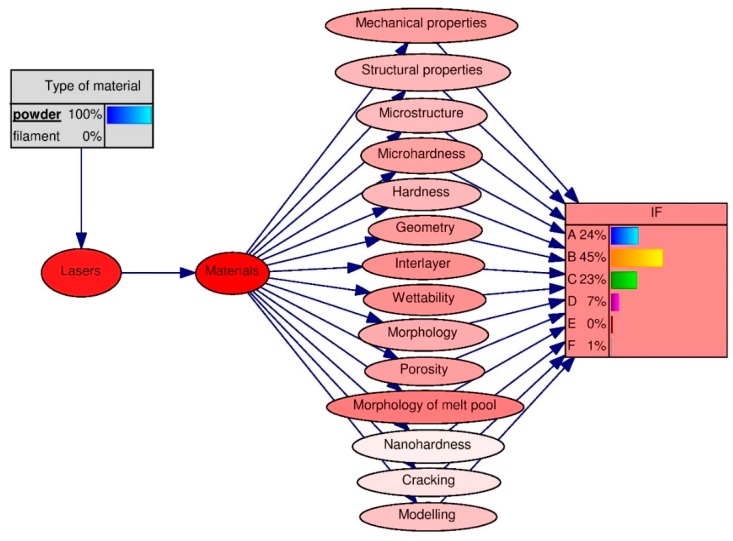
Expert knowledge levels of new technologies obtained via investigations of single-track features in laser processing, for studies performed using powder materials and the IF—the impact factor of the journal in which the research article was published. The level of expert knowledge in the journal with a specific IF index was: A = 24% (IF ≤ 2), B = 45% (2 < IF ≤ 3), C = 23% (3 < IF ≤ 4), D = 7% (4 < IF ≤ 5), E = 0% (5 < IF ≤ 6), F = 1% (IF > 6).

According to the accumulated expert knowledge regarding a single track in laser cladding, the knowledge-level values were obtained for each single, identifiable factor in the knowledge base:

- for material type: filament (Figure 7);
- for material type: powder (Figure 8).

The application of the Bayes algorithm (Figure 7 and Figure 8) to the knowledge base, in accordance with the designated learning Bayesian network (Figure 6), allowed the knowledge levels of the new technologies of single-track features in laser processing to be determined, for the studies performed on the following types of materials: filament and powder.

The intensity of the colors assigned to the properties tested for a given material type, i.e., the input parameter, indicates the degree of their mutual connection. Accordingly, in the studies where a filament was used (Figure 7), regardless of the material, the most common properties tested were the microstructure and hardness, and the least common were the structural properties. 

However, for powder (Figure 8), regardless of the material, the most common properties were the mechanical properties, geometry, interlayer, wettability, and features of the melt pool. Properties such as nano-hardness did not draw the interest of researchers. Moreover, it was found that for the level of knowledge of new technologies, using investigations of single-track features in laser processing as an example the highest-IF journals were marked as group B for the material types of filament (43%) and powder (45%). A low level of knowledge, for both the research conducted on the fiber type and the powder material, was determined for the periodicals in groups D, E, and F. 

Subsequently, using the Bayes algorithm, the dependencies among the tested materials were determined, as well as their properties, i.e., the mechanical properties, structural properties, microstructure, microhardness, hardness, geometry, interlayer, wettability, morphology, porosity, the features of the melt pool, nano-hardness, cracking, and modeling. For a given material or input parameter, according to the knowledge base and network model (Figure 5), it was found that in tests where a material such as an Fe alloy was used, research was most often performed on modeling and cracking, and the property tested was nano-hardness. The structural properties, hardness, and porosity were tested for Ti materials, while testing was not performed on the nano-hardness. For Cr–Ni, the largest area of research was tests of properties such as the microstructure, microhardness, morphology, porosity, and features of the melt pool. For ceramics, the most common areas were the microstructure, hardness, geometry, and morphology, while for the material mechanical properties, they were the microstructure, microhardness, hardness, geometry, morphology, porosity, and the features of the melt pool. In turn, for materials such as high-speed steel, it was determined that the researchers focused on cracking tests, and in the case of stainless steel, the researchers focused on the mechanical properties, microstructure, microhardness, geometry, interlayer, wettability, porosity, and features of the melt pool. Moreover, for bronze, most researchers investigated modeling. For materials such as special alloys, the following properties were tested: microstructure, micro-hardness, hardness, and morphology. No test properties were identified for tool steel. For superalloy materials, the most common properties were the geometry, morphology, and the features of the melt pool. 

The level of knowledge of new technologies, for research performed on a given material (Table 2), was determined.

The knowledge level of research on materials such as Fe and high-speed steel was highest for journals with an IF belonging to groups B and C, and that for Ti, Cr–Ni, Al, stainless steel, and special alloys was highest for journals in groups A and B. For ceramic materials, the level of knowledge was similar to that for journals in groups A and B (B: 39%, C: 38%, respectively). For bronze and superalloy materials, the highest knowledge level was for journals in group B, and for tool steel, the highest knowledge level was for journals in group C. 

According to the knowledge base and network model (Figure 5), research was conducted to determine the relationship between the laser type used for tests and the properties tested. It was found that in studies where fiber lasers were used, the mechanical properties of the materials were most often examined, while in the case of SLM machines, as well as Nd:YAG, the features of the melt pool were studied most frequently. When hybrid lasers were used, the following properties were investigated: the geometry, morphology, and the features of the melt pool. When a high-powder diode was used, the properties included the microstructure, hardness, and geometry. When a CO_2_ laser was used, the properties studied were the morphology and porosity, and when self-development machines were used, the structural properties were studied. Therefore, the levels of knowledge of new technologies were determined (Table 3).

The level of knowledge in the research conducted on all laser types was highest for the journals in group B. Similarly, a high level of knowledge (30%) of the research conducted on fiber lasers and self-development machines was determined for journals in group C (for fiber lasers) and group A (self-development machines). The next section provides the practical application of the knowledge received using the Bayes algorithm.

## 4. Experimental Procedure

It was said above that the other aim of this study is to analyze the possibility of depositing materials which were selected on the basis of the expert knowledge level. 

Based on the above-mentioned development trends in AM processes by using the Bayes algorithm, the following elements for the subsequent experimental part were determined:

- the type of material: powder, because a high level of knowledge of new technologies, using investigations of single-track features in laser processing, was defined for the powder material types (45%).
- the type of laser: self-development machine, because a high level of knowledge for such type of laser was determined in the journals of B group (IF 2 < IF < = 3), (Table 3).
- the material: composite material based on the Ni alloy and Fe–Al bronze; it is easy to see that the possibility of using Ni-based alloys and bronze in ADM is discussed in publications with the most significant IF factor (Table 2).
- the properties tested: mechanical properties, structural properties, interlayer, and wettability were chosen according to the study of trends. For powder (Figure 8), regardless of the material, the most common properties were: the mechanical properties, interlayer, and wettability. Moreover, we discovered that when self-development machines were used, the structural properties were most studied (Figure 5). In experimental research, the microhardness and microstructural properties were studied because they are most often studied according to the database that was developed.

### 4.1. The Self-Development Machine for Material Deposition 

The self-development machine was constructed basing on a continuous-wave CO_2_ laser “Comet 2” with the power of 1 kW. The lens of own design for coaxial discrete powder feeding was used. Three or more powders may be fed into the depositing zone. According to Zekovic et al. [59], such a design of the lens provides the possibility of depositing powders in any feed direction and there are fewer requirements concerning the powder distribution system.

**Figure 9:**
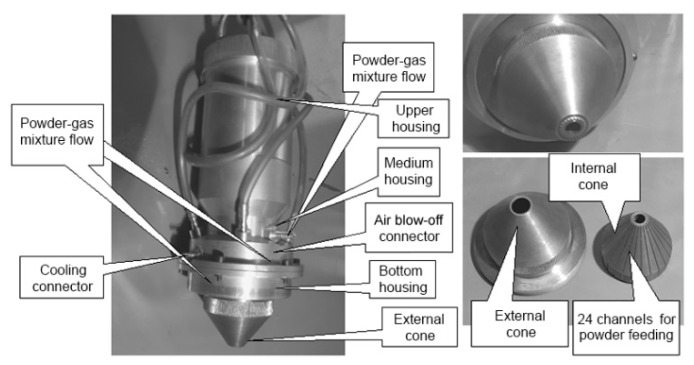
The principle scheme of laser lens.

The lens design is shown in Figure 9. The powder is fed into the expansion chamber of the nozzle, where a powder gas cloud is formed, which is distributed uniformly around the laser beam. The laser beam is focused by the laser lens depending on the focal length. The powder flow passes through the channels of the internal cone, which transforms it into a quasi-laminar flow parallel to the focusing axis of the laser beam. The ring groove of the nozzle cap focuses the powder flow into the laser spot on the surface being deposited. The additional gas feed from the blowing channel protects partially the melt pool against oxidation (if the process uses a material that requires processing in a protective environment). In continuous operation, the internal and external parts of the lens are cooled by air intensively.

The upper housing is a cylindrical part with two threaded connections. The upper thread is designed for fixation in the laser device and the lower thread is for connection to the medium housing. There is a groove for mounting the focusing lens inside the cylinder.

The middle housing is bolted to the lower housing. Special grooves are made on the connecting surfaces of the housings to form an internal ring-type channel when the housings have been joined. This channel is used for cooling air feed and is sealed with special seal rings. Additionally, four channels have been drilled in the middle housing conjointly with the bottom one for feeding the powder-gas mixture at an angle of 120 to the surface to be deposited.

The unit that directs the powder flow onto the surface to be deposited and forms this flow consists of an internal cone and an external cone. The internal cone has 24 channels through which the powder-gas mixture is fed directly into the laser beam area. Besides that, by rotating the external cone through the threads of the lower housing, the clearance between the cones can be additionally adjusted. It provides an additional powder feed along the cone’s elements in addition to the flow through the channels.

When the lower housing and two cones, external and internal, are connected together, a mixing chamber is formed between them. In this chamber, the powders are mixed and fed into the zone of laser action through the grooves in the internal cone of the lens. Additionally, a plate divider is installed in the mixing chamber, the displacement of which depends on the fraction of the powder used and its flow rate. If the divider is not configured, it is possible for powder particles to enter directly into the feed channels without being mixed qualitatively. The relative position of the two cones can also be adjusted. This is done by their mutual rotation in the lower housing. Thus, it is possible to adjust the clearance between them. This is necessary to compensate for possible manufacturing inaccuracies and to open or close an additional ring channel for powder feed if the flow rate needs to be increased. The position of the external cone is fixed after adjustment. When feeding the powder, an additional powder jet separator nozzle is used to separate the powder flow.

The lens designed can work in conjunction with a variety of powder-gas feeders and provides simultaneous or discrete feeding of up to four different powders at a minimum focal length of 100 mm and a maximum of 200 mm. It can form both round and rectangular laser spots. The inclination of the lens does not affect the powder feeding conditions.

### 4.2. Materials

The AISI 1045 steel, which is very popular in machine production, was used as the bottom substrate. A block of this steel was mounted on a laser platform and then single tracks were deposited. Two types of powders were used for the research: an 12N-01 Ni-based 12N-01 alloy (Table 4) and Al–Fe powder bronze (Table 5). In the initial state, the hardness of the Ni-based alloy was HRC 35-40 (HV 350-400) and the hardness of Al–Fe powder bronze was HRB 65-70.

The choice of materials for DMD processing is justified by the need to ensure a high-quality bonding between the materials of single tracks and the bottom substrate material.

Compressed air was applied as a transporting gas. The focal length was 200 mm and the laser spot diameter was 1.0 mm, which corresponds to a power density of 1.27–105 W/cm^2^. Earlier tests revealed [17,60] that optimal granulation of the powder tested was of 20–80 μm. The distance between the nozzle and the sample surface was of 10 mm.

### 4.3. Samples Preparation 

**Figure 10:**
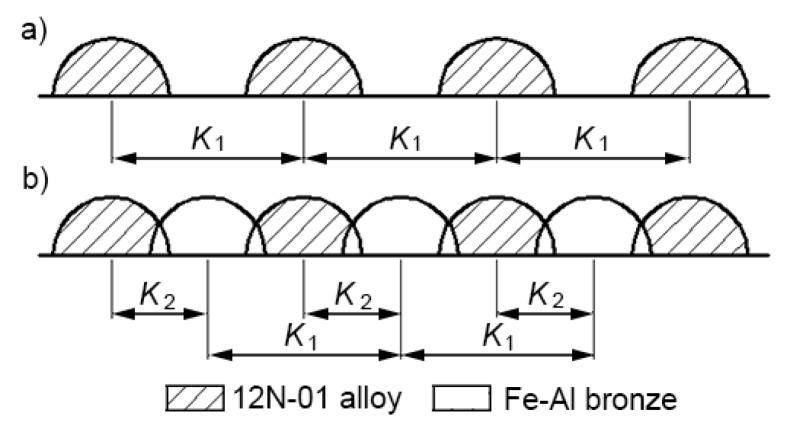
The scheme of depositing a single composite layer: (**a**) tracks of 12N-01 alloy; (**b**) layer of 12N-01 alloy and Fe–Al bronze.

Single and double composite layers were investigated. In the case of a single layer, parallel tracks of 12N-01 alloy were deposited first on the steel bottom with pitches K1 = 1.8; 2.4 and 3 mm (a), and tracks of Fe–Al bronze were deposited between them in the second step (b). The pitches between tracks of bronze were K2 = 0.9, 1.2, and 1.5 mm. The scheme of depositing a single composite layer is shown in Figure 10.

**Figure 11:**
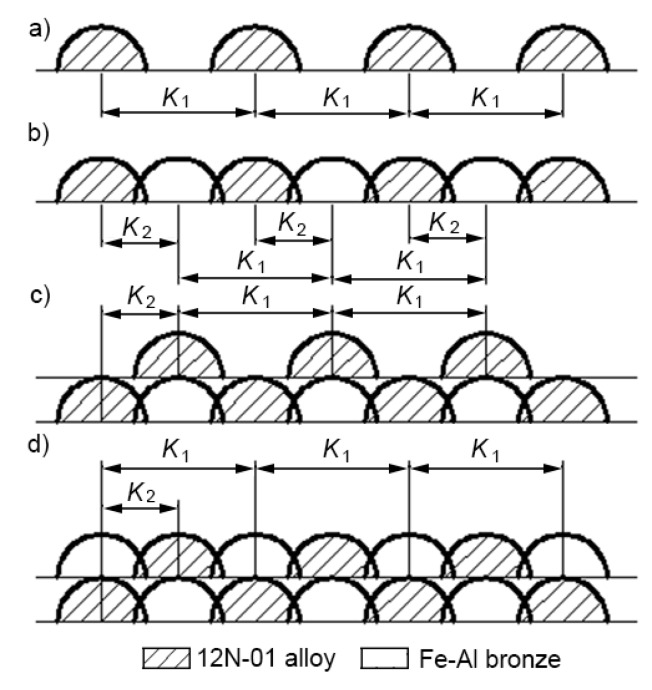
The scheme of depositing a double composite layer: (**a**) tracks of 12N-01 alloy on the steel substrate; (**b**) single layer of 12N-01 alloy and Fe–Al bronze on the steel substrate; (**c**) tracks of 12N-01 alloy repeated again; (**d**) double layer of 12N-01 alloy and Fe–Al bronze.

In the case of a double composite layer, depositing was performed in four stages (Figure 11). In the first stage (a), parallel tracks of the 12N-01 alloy were cladded on the steel bottom with pitches K1 = 2.0, 2.2, and 2.4 mm. In the second stage (b), tracks of bronze were deposited between them, and the pitches between tracks of the Ni alloy and bronze were K2 = 1.0, 1.1, and 1.2 mm. The formation of the first layer was completed at this stage. In the third stage (c), parallel tracks of the 12N-01 alloy were again deposited on the first layer, and these tracks were deposited with displacement relative to tracks of the first layer so that tracks of the 12N-01 alloy were located above tracks of bronze of the first layer, steps K1 between tracks of the 12N-01 alloy were 2.0, 2.2, and 2.4 mm. In the fourth stage (e), bronze tracks were deposited between the 12N-01 alloy tracks and pitches K2 between the Ni alloy and bronze tracks were 1.0, 1.1, and 1.2 mm. 

### 4.4. Measuring Equipment

The microhardness of the single tracks and layers deposited was measured using a Micromet II tester under the load of 100 gf. The average standard deviation for microhardness measurements was equal ~5%. The microstructures were investigated using a “Mira” scanning electron microscope of «Tescan» (Brno, Czech Republic). The graphical analysis of the results obtained was performed with the use of the Statistics 13 software.

### 4.5. DMD of Composite Layers

#### 4.5.1. Single Layers Deposition

**Figure 12:**
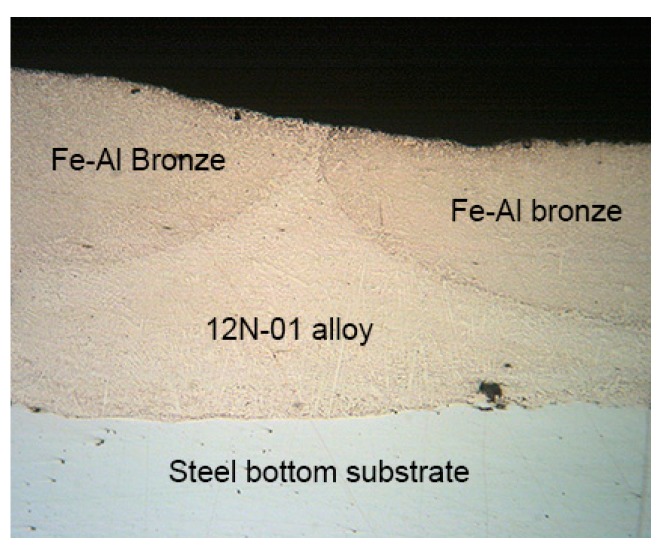
The cross-section of single composite layer (50x).

Two tracks of Fe–Al bronze, between which there is a track of 12N-01 alloy, can be seen in Figure 12.

**Figure 13:**
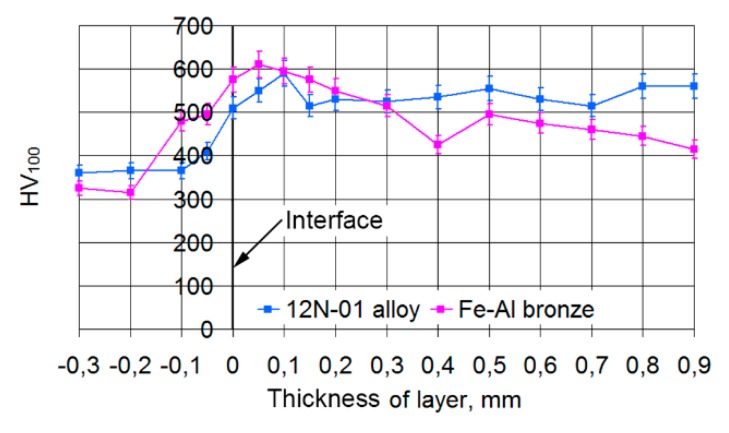
Microhardness distribution through the thickness of a single composite layer: the cladding speeds of alloy and bronze are, respectively, 120 and 200 mm/min; the cladding pitches of alloy and bronze are K1 = 2.4 mm and K2 = 1.2 mm, respectively.

The character of microhardness distribution through the thickness of a single composite layer is shown in Figure 13.

It can be seen in Figure 13 that there is a strong growth of microhardness at the interface between the steel and the deposited layer, which indicates that the transient zone between them is very small or absent. Furthermore, the microhardness of the 12N-01 alloy is approximately constant through the thickness of the layer. This is evidence of good mixing of the material in a bath of melt and uniform heating of the track material. The microhardness in the tracks of the 12H-01 alloy is HV100 = 550 on average.

Slightly different results were observed in a single track of Fe–Al bronze. On the interface between the steel and bronze, as well as in the tracks of the 12N-01 alloy, there is a sharp increase in microhardness, and, at a distance from the steel of 0.25 mm, the average value of microhardness in the track is HV100 = 550, that is equal to the microhardness of the 12N-01 alloy. Hereafter, the microhardness decreases to an average level of HV100 = 450 and stays at this level through the thickness of the layer. Hence, uniform heating and good mixing of a material in a bath of melt take place when depositing a bronze track. 

**Figure 14:**
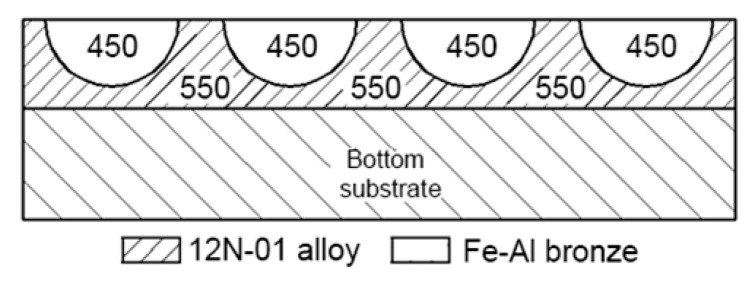
The scheme of microhardness changes in a single composite layer.

The character of microhardness change in a single composite layer is shown in Figure 14. Such a layer may be considered as a “matrix” of the 12N-01 alloy with average microhardness HV100 = 550, in which areas of bronze with average microhardness HV100 = 450 are embedded using laser deposition.

**Figure 15:**
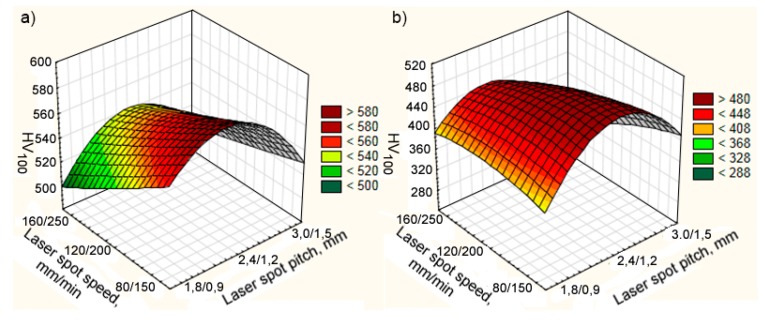
The influence of laser spot speed and pitch of laser deposition on microhardness of components in a single composite layer: (**a**) 12N-01 alloy; (**b**) Fe–Al bronze.

The influence of the speed and pitch of laser deposition on the microhardness of components in a single composite layer of 12N-01 alloy and Fe–Al bronze is shown in Figure 15.

The average microhardness of the 12N-01 alloy decreases as the cladding speed increases regardless of the laser spot pitch. At cladding speeds of Ni alloy and bronze VNi-al/Vbr = 80/150 mm/min, respectively, the energy contribution to the depositing layer is sufficient to form a relatively large melt bath. At the same time, the solidification rate is high enough to form a structure with increased microhardness. At a further increase of depositing speeds to VNi-al/Vbr = 120/200 and VNi-al/Vbr = 160/250 mm/min, the energy contained in a composite layer decreases. Respectively, the size of the melt bath decreases and the solidification rate increases when the same quantity of powder material is fed. As a result, the incomplete fusion of materials on the borders of single tracks becomes more and more significant. That leads to a decrease in the average microhardness of the 12N-01 alloy in the composite layer.

The effect of pitch on the average microhardness of the 12N-01 alloy is more complicated. The highest microhardness at all cladding speeds is observed at the cladding pitch of tracks K1 = 2.4 and K2 = 1.2 mm. In this case, due to good heating and cooling conditions of the neighboring tracks, favorable conditions are created for the formation of an optimal structure, which causes an increase in microhardness. When the pitch of the track decreases to K1/K2 = 1.8/0.9 mm due to repeated heating of the neighboring tracks, the material is overheated and the grain size increases. It leads to a decrease in microhardness. When the pitch is increased to K1/K2 = 3.0/1.5 mm, the reheating of the neighboring tracks will have little effect on their cladding. In this case, the incomplete fusion of the material along the grain boundaries starts exerting its effect and causes a decrease in microhardness of the 12N-01 alloy.

The average microhardness of bronze in a single composite layer is slightly dependent on the depositing speed. The influence of the laser spot pitch is similar to the effect it has on the 12N-01 alloy. A higher microhardness is observed at cladding pitches K1/K2 = 2.4/1.2 mm. At a decrease in pitch to K1/K2 = 1.8/0.9 mm and at an increase to K1/K2 = 3.0/1.5 mm, the microhardness of bronze decreases. The reasons for such changes are also due to changing heating and cooling conditions.

**Figure 16:**
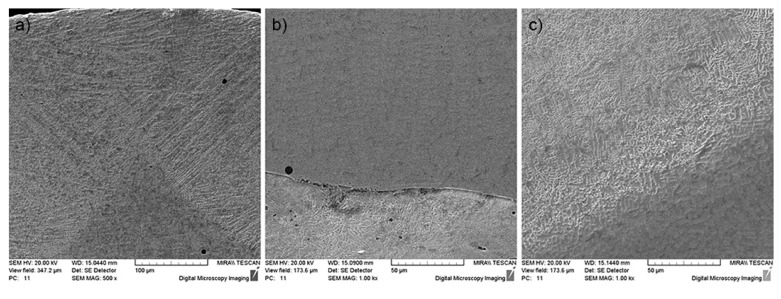
Microstructure of a single layer in different areas: (**а**) the track of the 12N-01 alloy in the transient zone between two tracks of bronze (VNi-al/Vbr = 120/200 mm/min, K1/K2 = 2.4/1.2 mm); (**b**) the transient zone between the tracks of the 12N-01 alloy and steel (VNi-al/Vbr = 120/200 mm/min, K1/K2 = 2.4/1.2 mm); (**c**) transient zone between the tracks of the 12N-01 alloy and bronze (VNi-al/Vbr = 160/250 mm/min, K1/K2 = 1.8/0.9 mm).

Bronze tracks deposited on the 12N-01 alloy tracks crystallize as dendrites with axes at an angle of 45° to the surface, which indicates a high rate of crystallization. The alloy has a globular structure in which the eutectic component is clearly distinguished (Figure 16a). As the cladding rate increases, the depth of the transient zone decreases (Figure 16b), and continuous iron solid solutions in nickel do not have time to form, but the chemical bond between components is high enough. As the cladding speed of both the Ni alloy and bronze increases, the size of the structural components decreases and the dendrites have only first order axes (Figure 16c), and transfer to a quasi-eutectic state.

#### 4.5.2. Double Layers Deposition

**Figure 17:**
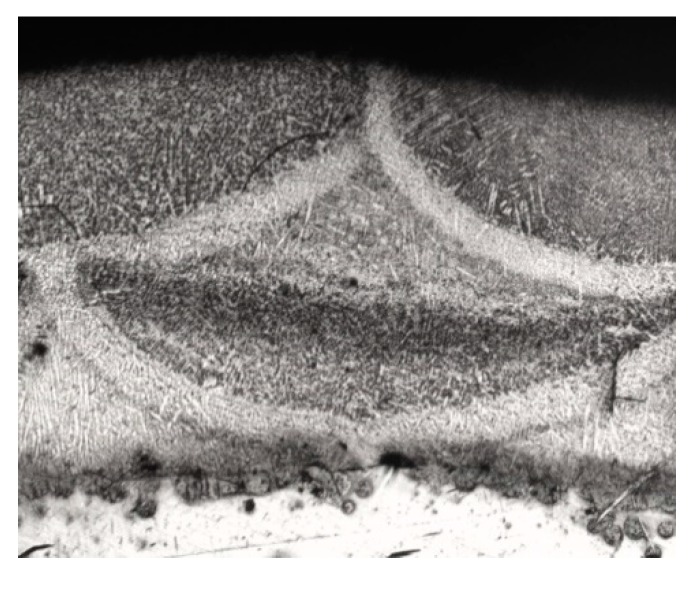
The cross section of a double composite layer (50 ×).

In Figure 17, in the first layer two tracks of the 12N-01 alloy are clearly visible, between which there is a track of bronze. In the second layer above the track of bronze of the first layer, there is a track of the 12N-01 alloy, to the right and left of which there are tracks of bronze.

**Figure 18:**
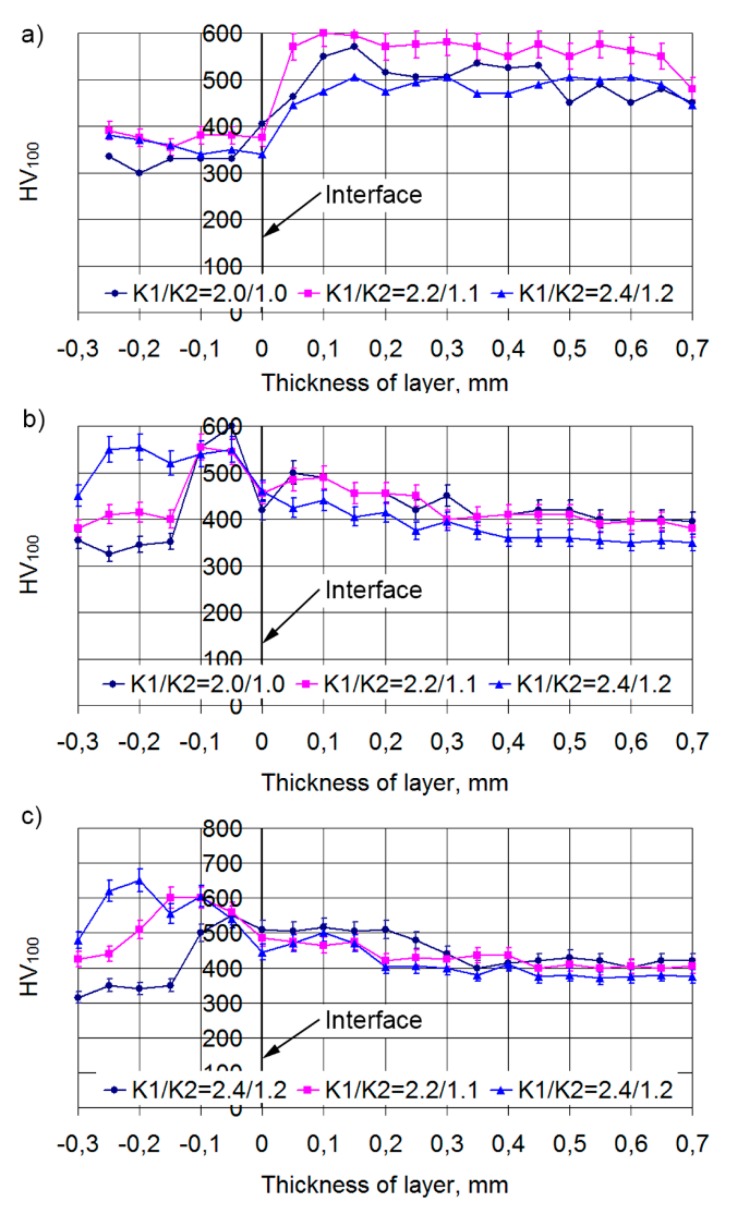
Microhardness distribution through the thickness of a double composite layer: (**a**) cladding speeds of Ni alloy/bronze VNi-al/Vbr = 100/150 mm/min; (**b**) cladding speeds of Ni alloy/bronze VNi-al/Vbr = 120/180 mm/min; (**c**) cladding speeds of Ni alloy/bronze VNi-al/Vbr = 140/210 mm/min.

The character of microhardness distribution through the thickness of the layer depends quite strongly on the laser spot speed. At low speed (VNi-al/Vbr = 100/150 mm/min) there is a strong overheating of a steel bottom and a sharp growth of microhardness to HV100 = 500−580 is observed on the steel-track border (Figure 18a) that corresponds to the data for the 12N-01 alloy. At this level, the microhardness is maintained to a distance of 0.5−0.95 mm from the steel bottom. After this, there is a decrease in the microhardness of the layer to HV100 = 420−470. This is connected with the transition of microhardness measurement direction to the bronze component of the composite layer. The transfer from one microhardness zone to another is accompanied by a rather sharp change in microhardness, which is usually due to the small depth of the transient zones.

When the depositing speed is increased to VNi-al/Vbr = 120/180 mm/min, there is a decrease in microhardness on the steel-layer border (Figure 18b). Then, the microhardness in the alloy zone within a distance of 0.2−0.3 mm from the steel surface is HV100 = 420−500. Furthermore, at transfer to a bronze component, microhardness decreases to HV100 = 370−420. As at transfer from one zone to another, there is no considerable difference in microhardness, it is possible to assume the presence of transfer zones between the 12N-01 alloy and bronze that are rather big in size.

It is visible at a cladding speed of VNi-al/Vbr = 140/210 mm/min (Figure 18c) that there is some decrease in microhardness on the steel-layer border. After this, microhardness in the alloy zone increases to HV100 = 470−530. Such microhardness is observed up to a distance from the steel base of 0.15−0.2 mm. At a greater distance from the steel base, in the bronze zone, the microhardness of the layer decreases to HV100 = 400−440. Thus, two microhardness zones associated with different components of the layer are also observed at a higher deposition thickness. The transition zones in this case are large enough, as evidenced by the smooth change in microhardness at transition from one zone to another.

**Figure 19:**
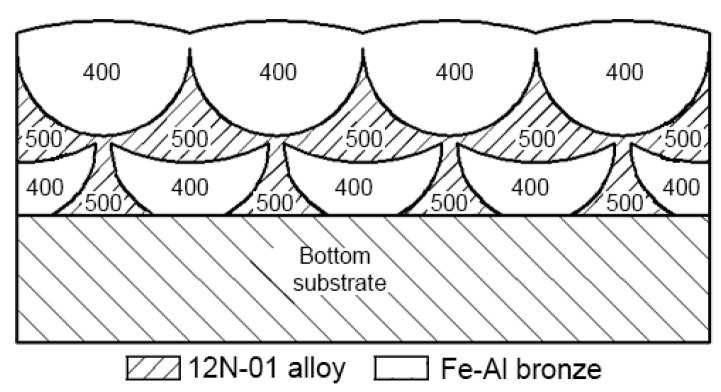
The scheme of microhardness changes in a double composite layer.

The observed periodicity of microhardness changes in the double composite layer is presented in Figure 19. Such a double layer represents a “matrix” which consists of a nickel alloy with average microhardness HV100 = 500, in which areas of bronze with average microhardness HV100 = 400 are embedded.

**Figure 20:**
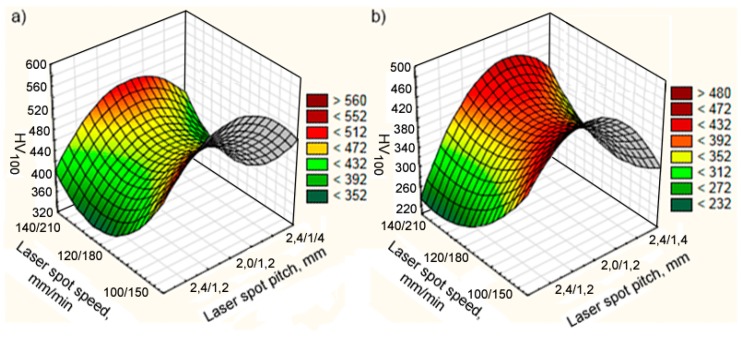
The effect of laser spot speed and pitch on the microhardness of the components of a double composite layer: (**a**) 12N-01 alloy; (**b**) Fe–Al bronze.

The influence of the speed and pitch of the laser spot on the microhardness of the components in the double composite layer of the 12N-01 alloy and Fe–Al bronze is shown in Figure 20. The complex nature of the impact of the parameters tested, similar to the case of a single composite layer, is caused by conditions of heating and cooling of the bath with melt. Regardless of the laser spot speed, the highest microhardness of the deposited layer is observed for spot pitches of 2.2/1.1 mm. However, in the average laser speed range, the microhardness of both nickel and bronze components is the lowest.

**Figure 21:**
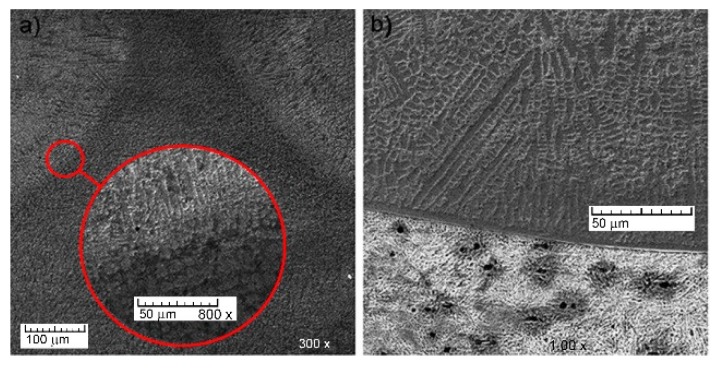
Features of the double layer formation: (**a**) junction of two bronze tracks with 12N-01 alloy track; (**b**) transfer zone between the alloy track and steel bottom.

The features of the microstructure of the double composite layer were investigated mainly in the areas of the nickel alloy and bronze interface (Figure 21). An intensive diffusion of layer components into each other due to their intensive mixing in the bath of melt was observed. It was found that nickel alloy and bronze areas in the double composite layer are very different in composition compared to the initial materials. Copper from bronze passes to the nickel alloy, and at the same time, Ni and Cr from the alloy diffuse intensely into bronze. Aluminum practically does not diffuse from bronze, but at the same time a significant diffusion of iron from the steel base into the nickel alloy and bronze of the molten layer is observed. It is important to emphasize the absence of defective zones, delamination, microcracks, etc., in the double composite layer.

## 5. Conclusions

In this paper, an extensive investigation into single-track features in SLM/SLS/DMD processing was conducted. The preliminary quantitative analysis of the knowledge and the Bayes algorithm as a representative of the data mining family were used to analyze development trends in the processes of additive manufacturing and then single and double composite layers were tested using the direct metal deposition of Ni-based alloy and Fe–Al bronze. Such an approach, namely, the combination of using the data mining techniques for the analysis of the scientific database of the phenomenon under study and subsequent experimental investigation into its features is the scientific novelty of the present study.

The Bayes algorithm is a very effective tool for assessing trends in the development of modern industrial technologies such as additive manufacturing. As a special example, the most dynamically developing SLM/SLS/DMD technologies were considered. Particularly, in determining the direction for further testing based on features of the material deposited, it was found that the following properties were tested to the smallest extent: mechanical properties, structural properties, microhardness, hardness, interlayer, and wettability. 

This analysis using the Bayes algorithm was structured according to the IF of journals contained in the knowledge base studied. It seems that in further research the parameter “the amount of citations” should be taken as the output for analyzing trends in new technologies, because it implicitly indicates the interest of both leading scientific journals and researchers in studying certain topics.

On the basis of the experimental research, features of composite layer forming were found. Particularly, in a single layer, a strong growth of microhardness at the interface between the steel substrate and layer deposited is observed, which indicates that the transient zone between them is very small or absent. The microhardness of the Ni-based alloy area is approximately constant through the thickness and is equal to HV100 = 550. The microhardness of the Fe–Al bronze area at first increases sharply at a distance from the steel of 0.25 mm and then stables to an average level of HV100 = 450. The microhardness of the single composite layer decreases as the depositing speed increases but the effect of the pitch is more complicated. In the single composite layer, bronze tracks contain dendrites, and Ni-based alloy tracks have a globular structure with the clearly distinguished eutectic component. As the depositing speed increases, the size of the structural components decreases and the structure becomes the quasi-eutectic.

In the double composite layer, the character of microhardness distribution through the thickness depends quite strongly on the laser spot speed. Such a layer represents a “matrix” consisting of a nickel alloy with microhardness HV100 = 500 in which areas of bronze with microhardness HV100 = 400 are embedded. The influence of the speed and pitch of the laser spot on the microhardness of the double composite layer components has a complex nature similar to the single layer. In the areas of the Ni-based alloy and bronze interface of the double composite layer an intensive diffusion of layer components into each other was observed. Compared to the initial materials, nickel alloy and bronze areas in the double layer are very different in composition. Copper from bronze passes to the nickel alloy, and at the same time Ni and Cr from the alloy diffuse intensely into bronze. The absence of defective zones, delamination, microcracks, etc., in the double composite layer is observed, which indicates the correct choice of initial materials for the generation of a composite material using the DMD method.

In summary, to guarantee good properties of the boundary area between the base metal plate and composite DMD volume, the composite part should have similar compounds. Such compounds improve their mutual binding in horizontal as well as vertical directions. In order to conduct research experiments, first, the current trends in the research using the Bayes algorithm should be determined and then AD technologies can be effectively applied in practice.

## Figures and Tables

**Table 1:** Conditional probability tables CPT for X2: laser.

Laser Types	Material Types	
	Powder	Filament
SLM machines	0.1792	0.0000
Nd:YAG lasers	0.1574	0.0000
CO_2_ lasers	0.0634	0.0000
High-power diode lasers	0.1206	0.6518
Self-development machines	0.1420	0.0000
Hybrid lasers	0.0452	0.0000
Fiber lasers	0.2923	0.3482

**Table 2:** Knowledge levels of new technologies based on the Bayes algorithm (factor: material).

Material	Expert Knowledge Levels of New Technologies Based on the Bayes Algorithm					
	Research Article A	Research Article B	Research Article C	Research Article D	Research Article E	Research Article F
Fe	0%	39%	41%	20%	0%	0%
Ti	31%	49%	11%	6%	0%	3%
Cr–Ni	27%	44%	19%	8%	0%	2%
Ceramic	16%	39%	38%	6%	0%	0%
Al	30%	43%	22%	5%	0%	0%
High-speed steel	0%	69%	30%	0%	0%	0%
Stainless steel	29%	45%	17%	7%	0%	2%
Bronze	0%	57%	14%	29%	0%	0%
Special alloy	38%	48%	10%	4%	0%	0%
Tool steel	0%	0%	100%	0%	0%	0%
Superalloy	16%	51%	22%	9%	0%	2%
Others	0%	0%	100%	0%	0%	0%

**Table 3:** Knowledge levels of new technologies based on the Bayes algorithm (factor: laser type).

Laser Type	Expert Knowledge Levels of New Technologies Based on the Bayes Algorithm					
	Research Article A	Research Article B	Research Article C	Research Article D	Research Article E	Research Article F
Fiber laser	20%	41%	32%	5%	0%	1%
SLM machine	27%	47%	18%	7%	0%	1%
Nd:YAG	26%	48%	17%	7%	0%	2%
CO_2_ laser	28%	46%	16%	7%	0%	3%
High-powder diode	18%	44%	27%	11%	0%	1%
Self-development machine	30%	47%	15%	6%	0%	2%
Hybrid fiber laser	16%	51%	22%	9%	0%	2%

**Table 4:** The chemical composition of the Ni-based alloy.

C, %	B, %	Si, %	Cr, %	Fe, %	Ni, %
0.3–0.6	1.7–2.5	1.2–3.2	8–14	1.2–1.3	The rest

**Table 5:** The Fe–Al bronze chemical composition.

Al, %	Fe, %	Cu, %
8.5–10.5	4	The rest
